# Factors Affecting Student Satisfaction With an Undergraduate Research Project

**DOI:** 10.1111/eje.70050

**Published:** 2025-09-24

**Authors:** Joseph Phizackerley, Ruby Long, James Field

**Affiliations:** ^1^ Cardiff University School of Dentistry Cardiff UK

**Keywords:** dental, education, project, research, undergraduate

## Abstract

**Aim:**

The aim of this research paper was to explore Bachelor of Dental Surgery (BDS) undergraduate student views of a research project (RP) that comprises part of the curriculum at Cardiff University (CU), Wales.

**Method:**

4th and 5th year students undertaking the RP were issued an anonymous cross‐sectional survey. 66 responses were obtained, giving a response rate of 52%. The questionnaire consisted of Likert‐style items, a satisfaction score and free‐text responses. Jamovi and Excel were used to conduct statistical analysis (multiple linear regression, *t*‐tests and Cronbach's alpha) of the results along with thematic analysis of the free‐text responses.

**Results:**

Students generally had a positive response to the RP and felt that the RP helped to improve their research skills. The responses showed a positive correlation between satisfaction and student input into the project topic (estimate = 0.84, *p* = 0.03), understanding of the purpose of the RP (estimate = 0.69, *p* = 0.023), perceived improvement of research skills (estimate = 0.97, *p* < 0.001), and how prepared students felt to begin their project (estimate = 0.92, *p* = 0.004). There were, however, areas of the RP which students found challenging, such as the balance of completing the RP alongside clinical time, as well as arranging timely interactions with supervisors.

**Conclusions:**

Overall, students had a positive outlook on the RP, with students who felt more prepared to begin the project and those who felt the project had improved their skills perceiving the project more favourably. This paper provides some insight into how such a project could be improved, for example, considering an increased timeline or a group format of the project along with future research directions.

## 1 Introduction

It was as early as 1926 that the Gies Report recommended that dental education should provide students with research opportunities [1], and then in 1990 the General Dental Council (GDC) published guidelines recommending that ‘teaching should introduce the student to the principles of scientific thought and argument including the evaluation of scientifically established facts, experimental design, statistics and the analysis of data and place the clinical instruction in the scientific context’ [2]. Not only do current dental education standards such as the GDC's preparing for practice document [3] and the Graduating European Dentist (GED) publication state the importance of research, but more widely, one of the pillars of excellence (clinical governance) is ‘evidence based care and effectiveness’ [4], which requires a good understanding of how to conduct and evaluate research. The GDC stipulates that upon graduation, dental students should be able to utilise the principles of evidence‐based care in both learning and clinical and professional practice. This is in addition to being able to ‘evaluate evidence‐based prevention and apply appropriately’ [3]. The GED objectives also state that a graduating dentist must be able to ‘evaluate published clinical, scientific and public health related research and integrate this information to improve the oral health of the patient’ [5]. In addition to helping prepare students for practice, research can also help to guide students on an academic pathway in their career. It is therefore critical that undergraduate curricula give dental students the opportunities to develop and utilise these skills.

Recent European research [6, 7] shows that dental students know that they are required to understand how to conduct and interpret research—and a similar study conducted among US dental students showed similar results, that ‘dental students appreciate the importance of science and evidence–based practices in dentistry’ [8]. Ultimately, the Bachelor of Dental Surgery (BDS) curriculum is designed to equip students with the skills they will require in practice to provide the best patient care. This care should be based on current evidence [9], which requires the use of data from multiple sources to determine the most appropriate course of action. This highlights the importance of students developing these skills not only to pass exams but to utilise them during their time as a practising dentists.

**FIGURE 1:**
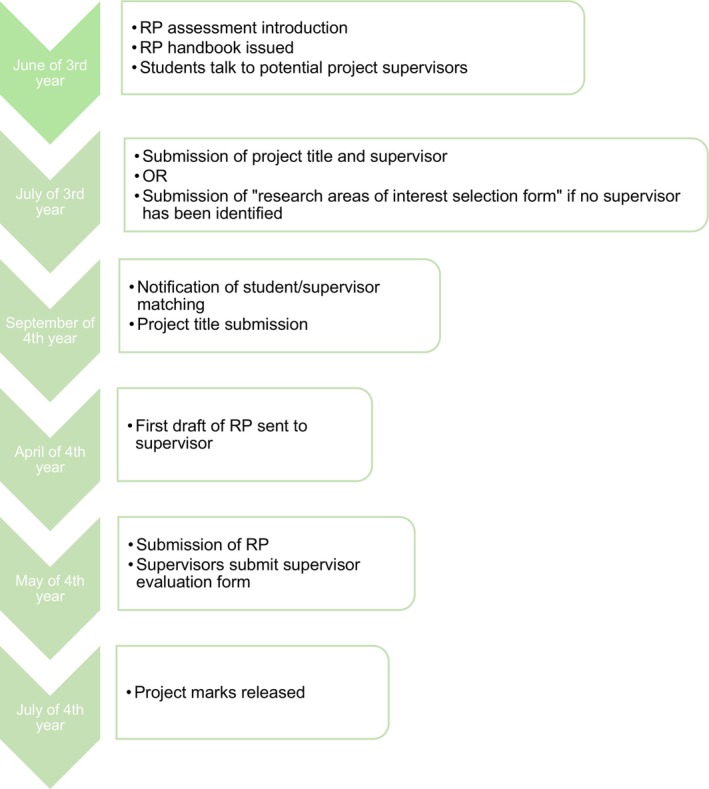
The timeline of completion of the project at Cardiff University.

The ability of BDS students to evaluate and conduct research is assessed at multiple points through the undergraduate degree at CU (Cardiff University), including completion of a research project (RP). The project is assessed summatively, with the topic of study usually being student‐led. Supervisors can be clinical or non‐clinical members of staff. The project currently is completed over 11 months (see Figure 1) and culminates in the production of a report of 4000–6000 words; however, the project was previously completed in the fifth year of study with a longer timeline. This project comprises 9% of the final degree classification, so it is important that students are able to effectively conduct and analyse research. The RP alone allows students to meet the majority of GED Domain V learning outcomes [7], and along with other components of the course, enables students to hone their understanding of research.

Existing literature suggests that students who have engaged in scientific research during their time at university tend to perform better academically [10, 11]. Dissertations play an important part of many undergraduate degrees, but there is very little research into the utilisation of a research project as part of an undergraduate dentistry degree, particularly in the UK. This could be due to the limitations in the assessment of essays and similar projects, which include reliability being low, labour‐intensive marking, a high risk of examiner bias and low validity in testing higher‐order cognition [12]. In addition to this, the current ease of access to artificial intelligence, which can be utilised to aid in writing or other aspects of project completion, may lead to these projects not being a true reflection of students' abilities.

This study aims to explore student views of the RP at CU. The objectives are to determine students' perceived preparedness to complete the RP, to determine students' opinion of the importance of the RP, and to determine whether students or supervisors are driving the RP process. The hypotheses of this project are that:

– As students progress through the course, the perceived benefit of the RP will increase.
– Students who feel more prepared to start the RP are more likely to drive interactions with their supervisor and to determine the project title.
– Students who feel better prepared are more likely to perceive a benefit to the RP.

## 2 Methodology

### 2.1 Data Collection

This cross‐sectional study was approved by the Cardiff University Dental School Research Ethics Committee on the 3rd November 2023 (project number 2330a).

The research of this project was conducted using survey methods, which formed a piloted questionnaire comprised of 8 items, Appendix A. This questionnaire was developed on an ad hoc basis without previous instruments; however, it was designed to assess different factors that may impact on student experience with their RP. These factors include student‐supervisor interactions as well as preparation for the project. These items took multiple forms, including Likert‐type five‐point scale items, multiple choice items and free text answers. Within the questionnaire, item order was randomised, but the Likert items encompassed three main themes:

– Perception of requirement for the project and benefit of the project
– Perceived preparation for the project
– Interaction with supervisors

The Likert items were followed by a ranking of project experience and three open‐ended items asking participants about their experience with the project, what they had learned from the project and about barriers/facilitators to completing the project. There was also an item asking participants if they had previous research experience; the items were designed to allow students to express their opinions on the RP in a variety of ways. Convenience sampling was utilised to recruit students to complete the survey, with all students on the BDS programme in 4th or 5th year during 2023/2024 being eligible to participate in the research. The aim was to recruit all students in these years (around 160 students) to complete the survey. An email was communicated to students with a link to complete the questionnaire, along with a participation information sheet which gave information on the questionnaire, with a follow‐up email being sent midway through the data collection period to encourage completion of the questionnaire. The questionnaire was open for 3 months (November and December 2023 and January 2024) with anonymous data being collected electronically via Microsoft forms.

### 2.2 Statistical Analyses

Statistical analysis was conducted on the quantitative data using both Jamovi version 2.3 and Excel version 2403. Prior to analysis, the answers to Likert item 11 (I had no input into the project title) were inverted to enable analysis by making the positive responses equivalent to a higher score in line with other items. Excel was used to calculate Cronbach's Alpha to indicate the internal consistency of the survey. The value was found to be 0.77, giving an adequate level of internal consistency [13]. Jamovi was utilised to conduct a principal component analysis (PCA). This PCA identified two main groups of Likert questions, which were questions regarding interactions with supervisors (‘I, rather than my supervisor, decided the focus of my RP’, ‘I have had regular interactions with my supervisor’, ‘My supervisor drove the method of interaction and organised our meetings’, ‘I had no input into the project title’ and ‘I arranged meetings with my supervisor’) and the remaining questions. The authors identified two separate themes of the remaining questions, which were further divided into questions assessing perceived preparation for the project (‘I felt enough information was given prior to beginning the RP, e.g., guidance, resources etc.’, ‘I have had other opportunities within the curriculum to undertake research’, ‘I feel/felt adequately prepared to begin my RP’) and perception of the requirement of the project and benefit of the project (‘I understand the purpose of the RP’, ‘I think the research project will benefit me upon completion of my undergraduate degree’, ‘I understand why the project forms part of my education’ and ‘I feel the RP has helped me develop my research skills’).

Jamovi was used to calculate the mean satisfaction score of both 4th and 5th year students and then conduct an independent *T* test to check for a significant difference. Jamovi was also used to calculate multiple linear regression (MLR) to assess which aspects were the best predictor of students' satisfaction as well as a correlation matrix of the responses to the Likert‐style items and the satisfaction score. To conduct this analysis, the statements strongly agree, somewhat agree, etc. were converted into a numerical value with strongly agree = 5, somewhat agree = 4, neither agree nor disagree = 3, somewhat disagree = 2 and strongly disagree = 1. The MLR results were simplified to contain only the main terms as well as significant interactions of year group. Qualitative data from the free text responses were reported as part of thematic analysis [14]. The data were not coded, but the emergent themes were manually determined by the authors following the consideration of participant responses. These identified themes were not further validated.

## 3 Results

### 3.1 Quantitative Results

There were 66 responses to the questionnaire, with 29 respondents in their 5th year of study and 37 respondents in their 4th year. This is a response rate of 44% for 5th years and 60% for 4th years. The students were asked to rate their experience with the RP on a scale of 1–10, with 1 being poor and 10 being excellent. The mean of these scores was 5.58, with a standard deviation (SD) of 1.9 and a median of 6.

**FIGURE 2:**
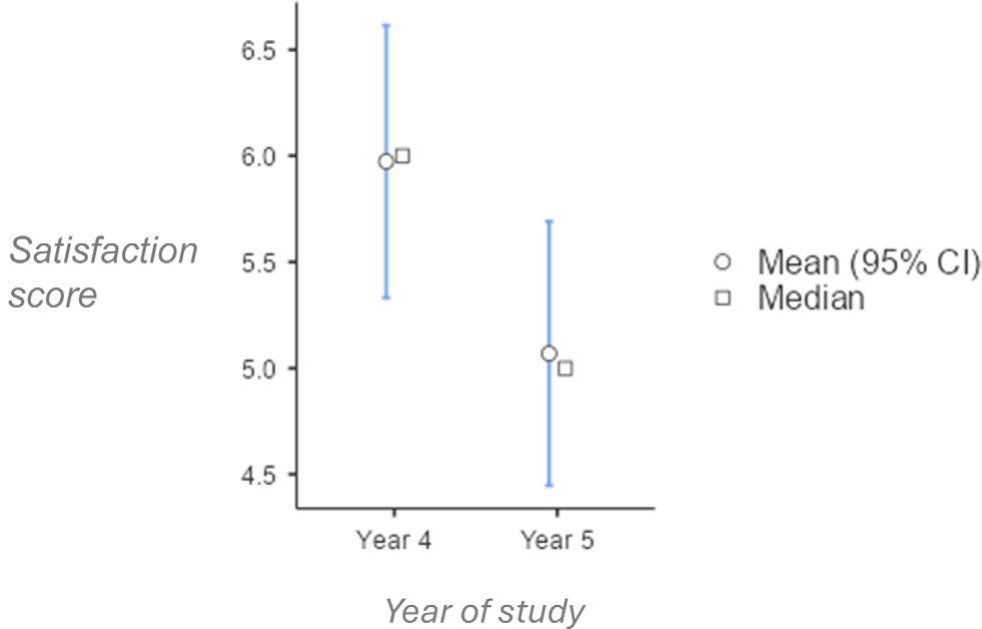
The mean and median satisfaction scores of 4th year students compared to 5th years among Cardiff University undergraduate students.

The mean of 4th year students' satisfaction scores was 5.97 (SD = 1.99) compared with 5.07 (SD = 1.71) for 5th year students; this is demonstrated in Figure 2. However, the independent samples *T*‐Test showed this difference to not be statistically significant (*t* = 1.94, df = 64, *p* = 0.056).

**FIGURE 3:**
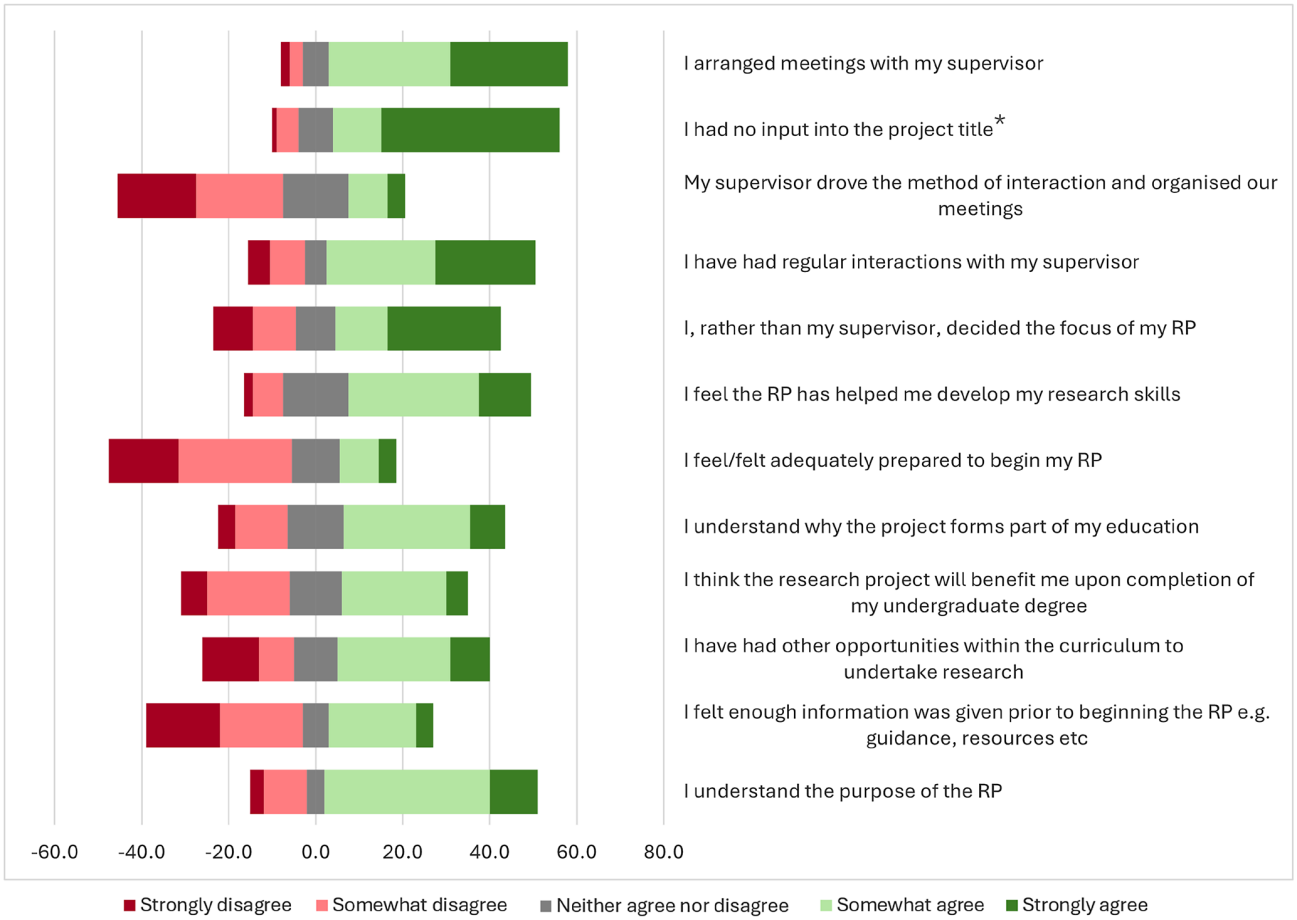
Likert plot to show the responses of Cardiff University students to Likert items on their opinions of the research project. *Responses to this question have been inverted to show positive responses in green.

The distribution of responses to the Likert items from students is shown in Figure 3. Students generally agreed that they:

- understood the purpose of the RP within their education,
- had other opportunities within the curriculum to undertake research,
- think the RP will benefit them upon completion of their undergraduate degree,
- understand why the RP forms part of their education,
- feel the RP has helped them develop their research skills,
- rather than their supervisor, decided the focus of their RP, and
- had regular interactions with their supervisor and that they arranged meetings with their supervisors.

Whilst the students tended to disagree that their supervisor drove the method of interaction, they largely disagreed with feeling adequately prepared to begin a RP and that enough information was given prior to beginning the RP.

### 3.2 Quantitative Results by Theme

The items were grouped into three themes following PCA:

1. Perception of requirement for project and benefit of project
  - ‘I understand the purpose of the RP’
  - ‘I think the research project will benefit me upon completion of my undergraduate degree’
  - ‘I understand why the project forms part of my education’
  - ‘I feel the RP has helped me develop my research skills’
2. Perceived preparation for project
  - ‘I felt enough information was given prior to beginning the RP, e.g., guidance, resources etc.’
  - ‘I have had other opportunities within the curriculum to undertake research’
  - ‘I feel/felt adequately prepared to begin my RP’
3. Interaction with supervisors
  - ‘I, rather than my supervisor, decided the focus of my RP’
  - ‘I have had regular interactions with my supervisor’
  - ‘My supervisor drove the method of interaction and organised our meetings’
  - ‘I had no input into the project title’
  - ‘I arranged meetings with my supervisor’

#### 3.2.1 Theme 1: Perception of Requirement for Project and Benefit of Project

Of the 66 responses, 64% (42 students) agreed that their project had improved their ability to conduct research or improved the skills required for the project, whilst only 14% (9 students) disagreed that the project had helped develop their research skills (Figure 3). While 44% of students (29 students) agreed that the project would benefit them after completing their undergraduate degree, 38% of students (25 students) did not think that the project would benefit them.

**FIGURE 4:**
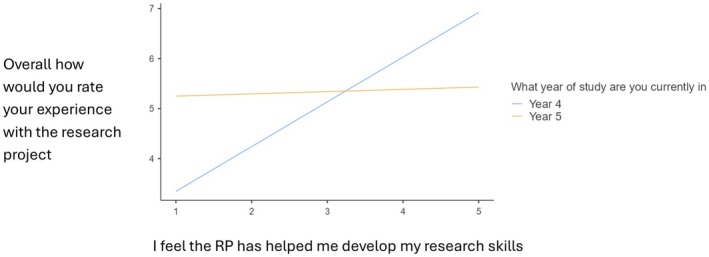
Estimated marginal means showing how student perception of how much the research project has helped develop research skills impacts on project experience separated into year groups.

The results of MLR analysis of Theme 1 items are shown in Table 1. The best predictor of student satisfaction with a RP (Table 1) was whether the students felt that the RP had helped develop their research skills (estimate = 0.90, *p* < 0.001) whilst the second best was students' thoughts on whether the RP will benefit them after graduation (estimate = 0.65, *p* = 0.002) followed by whether students understood the purpose of the RP (estimate = 0.45, *p* = 0.032). Students' understanding of why the RP forms part of their education (estimate = 0.11, *p* = 0.60) did not predict satisfaction scores. The adjusted *R* ^2^ value for these Likert items was 0.593 indicating that 59.3% of variability in student satisfaction could be explained by these variables. There was a statistically significant interaction between year of study and whether the students felt the RP helped them develop their research skills. Figure 4 shows the estimated marginal means for both groups, showing that among Year 4 students, their satisfaction improved as their sense of how much the RP helped them develop research skills increased, but for Year 5 students there was no relationship between the two variables.

**TABLE 1:** Results from multiple linear regression analysis of responses from Cardiff University students to Likert‐style items about the requirement of a research project and the benefit of the project and how these factors affect their satisfaction with the project.

Predictor	Estimate	SE	*t*	*p*	Adjusted *R* ^2^
I understand the purpose of the RP	0.45	0.20	2.20	0.03	0.593
I think the research project will benefit me upon completion of my undergraduate degree	0.65	0.21	3.19	0.002	
I feel the RP has helped me develop my research skills	0.90	0.24	3.72	< 0.001	
I understand why the project forms part of my education	0.11	0.21	0.53	0.60	
I feel the RP has helped me develop my research skills (Year 5–Year 4)	−0.85	0.31	−2.75	0.008	

#### 3.2.2 Theme 2: Perceived Preparation for Project

The second theme of Likert items was on the perceived preparation for the project and availability of resources. The results of MLR for these items are shown in Table 2. The best predictor of student satisfaction in these items was whether students felt adequately prepared to begin the project (estimate = 1.13, *p* < 0.001). Whether students felt enough information was given prior to the RP (estimate = −0.10, *p* = 0.65) and whether students have had other opportunities to undertake research within the curriculum (estimate = 0.11, *p* = 0.44) were not shown to be a good predictors of student satisfaction.

**TABLE 2:** Results from multiple linear regression analysis of responses from Cardiff University students to Likert‐style items about the students' perceived preparation for the research project and how this affects their satisfaction with the project.

Predictor	Estimate	SE	*t*	*p*	Adjusted *R* ^2^
I felt enough information was given prior to beginning the RP, e.g., guidance, resources etc	−0.10	0.21	−0.45	0.65	0.410
I have had other opportunities within the curriculum to undertake research	0.11	0.14	0.77	0.44	
I feel/felt adequately prepared to begin my RP	1.13	0.24	4.70	< 0.001	

The adjusted *R* ^2^ value for this MLR was 0.41 showing 41% of variability in student satisfaction could be explained by these variables. 55% of students (36 students) who responded thought that not enough information was given prior to beginning their RP and only 20% of students (13 students) felt like they were adequately prepared to begin the project (Figure 3). 53% of students (35 students) agreed that there had been other opportunities within the curriculum to undertake research.

Linear regression was used to determine if the students' opinions of whether they were prepared to start their RP could predict their perception of whether an RP would benefit them. The result of this linear regression (Estimate = 0.523, SE = 0.104, *t* = 5.02, *p* ≤ 0.001) shows that how prepared students feel may be able to predict their opinion of whether the RP will benefit them.

#### 3.2.3 Theme 3: Interaction With Supervisors

The final theme of the Likert items was how students interacted with their supervisors, with the results from these items showing that 83% of students (55 students) agree that they were the ones to organise meetings with their supervisors and 73% of students (48 students) had regular interactions with their supervisors. In addition to this, 79% of students (52 students) felt that they had some input into their project title, and 58% (38 students) of students felt that they were the ones to decide the focus of their project rather than their supervisor.

**FIGURE 5:**
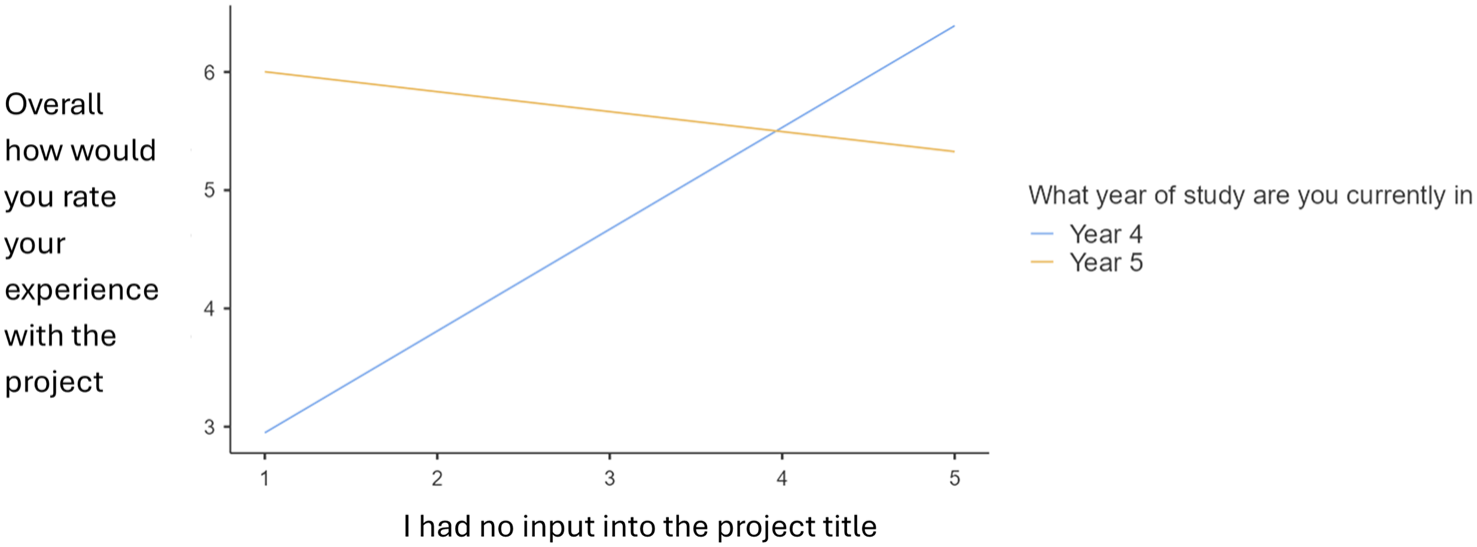
Estimated marginal means showing impact of input into project title on overall project experience separated by year of study.

Multiple regression analysis, Table 3, of these items showed that the best predictor of satisfaction score was whether or not students had input into the project title (estimate = 0.86, *p* = 0.004). Whether or not students arranged meetings with their supervisor (estimate = 0.42, *p* = 0.10), the regularity of meetings with supervisor (estimate = 0.43, *p* = 0.05), whether or not students decided the focus of the project (estimate = 0.01, *p* = 0.96) and if supervisors drove the method of interaction (estimate = 0.22, *p* = 0.30) was not shown to affect satisfaction score (Table 3). The adjusted *R* ^2^ value for this MLR was 0.404 showing 40.4% of variability in student satisfaction could be explained by these variables. There was a statistically significant interaction between input into project title and student satisfaction with the project. For Year 4 students there was a strong positive correlation between input and satisfaction but for Year 5 students there was a weak negative (Figure 5).

**TABLE 3:** Results from multiple linear regression analysis of responses to Likert items about Cardiff University undergraduate student views on interaction with supervisors during their completion of their project and how this affects their satisfaction with the project.

Predictor	Estimate	SE	*t*	*p*	Adjusted *R* ^2^
I, rather than my supervisor, decided the focus of my RP	0.01	0.18	0.05	0.96	0.404
I have had regular interactions with my supervisor	0.43	0.21	2.03	0.05	
My supervisor drove the method of interaction and organised our meetings	0.22	0.22	1.04	0.30	
I had no input into the project title^a^	0.86	0.29	2.98	0.004	
I arranged meetings with my supervisor	0.42	0.25	1.67	0.10	
I had no input into the project title^a^ (Year 5–Year 4)	−1.03	0.42	−2.46	0.02	

^a^ Scores for this item have been inverted.

### 3.3 Qualitative Analysis by Theme

From the free text responses, a number of themes were identified which included:

– Interactions with supervisors
– Lack of preparation and/or resources
– Problems with the time frame of the project
– Lack of participation in surveys
– Difficulties obtaining ethical approval
– Improved understanding of research and associated skills

#### 3.3.1 Theme A: Interactions With Supervisors

In the free text response items, 9 students (13%) left comments relating to the poor experience with supervisors. However, there were also 7 students (11%) commenting on positive interactions with supervisors with quotes being shown in Table 4. The students who left comments remarking on their negative interactions with supervisors had an average satisfaction of 4.67 compared with a score of 5.29 for those who commented on positive supervisor interactions.

**TABLE 4:** Quotations from a selection of fourth and fifth years discussed in the results.

	Please type three words which reflect your experience with the RP	What have you learned from completing/working on this project?	How do you think this has informed your future practice or learning?	What were/are the barriers and facilitators to you completing this project?
A	Stressful, confusing, challenging	How much is involved in a research project & how long it takes	If I need to search stuff in the future I think I will be in a better place to carry out research	Difficulties meeting with supervisors, different ideas to supervisors
B	Stressful, challenging, insightful	How to conduct your own research	Not informed it in any way other than conducting research	Difficulty contacting supervisor and gaining support. No support on audits from the library team
C	Stressful, time‐consuming, confusing	I have learnt a lot about my question title and how to structure a paper	It has given me lots of knowledge but about a niche topic	Availability of my supervisors—they often wanted to talk over email which made discussions very difficult and long and have lengthened the process out for me unnecessarily
D		How to carry out an audit, methods of carrying out your own research project	Will be useful for postgrad as I would like to specialise as I plan to publish and this is a step in the right direction to get into postgraduate specialist training	Not really any barriers, my supervisor is very helpful and the project has been a good experience so far
E	Stressful, fine, interesting	I have learnt how to go about developing a project, however it is difficult to manage time to ensure that the project is going well alongside other studies	I have developed an understanding of how to make improvements to a service, which could be useful when reflecting on my own practice in the future	Finding time alongside busy clinics and developing my understanding through studying has been difficult. Having a supervisor who is engaged and willing to help has been really helpful in my progress through the project
F	Unhelpful, futile, stressful	How to structure a research paper	I do not want to go into research	Facilitators—amazing and helpful supervisor. Barriers—word count
G	Challenging, stressful, interesting	How to conduct systematic reviews, how to critically appraise articles	Stronger ability to evaluate strength of evidence	Poor understanding of statistics, poor ability to write academic articles
H	Tedious, stressful, ok	Nothing new	Know how to structure a report	Lack of information on what is expected
I		I don't want to pursue academics	I don't feel like I've learnt much	Had no idea how to write a research paper
J	Confusion, overwhelming, interesting		Sparked interest to undertake more self‐directed research in the future	Unawareness of where to begin, limited understanding of how to conduct research
K	Confusion, stress, pointless	Improving organisation skills, learning how to research	It has helped me look into the area of dentistry that I am most interested in more, thus giving me more of an insight into the career path	I feel as though I have not been properly taught how to write a research project and could have done with some more input prior
L	Stressful, demanding, frustrating	How to write a literature review	May help if I was to undertake further study or go into academia. I don't feel it will help my clinical skills	Barriers: little information on how to write it, deadline too close to finals exams.
M		How to carry out a literature review	Helped understand how to research	Time with so many other deadlines
N	Self‐directed, confusing, long	Understanding what a literature review actually was	Ability to search research databases if I were to complete future research	Short time frame put me off doing my own primary research
O	New, winging‐it, hoping‐for‐the‐best	How to run systemic searches, how to assess the quality of studies	Introduction to research/writing	Not enough time to get ethical approval for primary data—I would have liked to have done this but couldn't due to time constraints

#### 3.3.2 Theme B: Lack of Preparation and Resources

**FIGURE 6:**
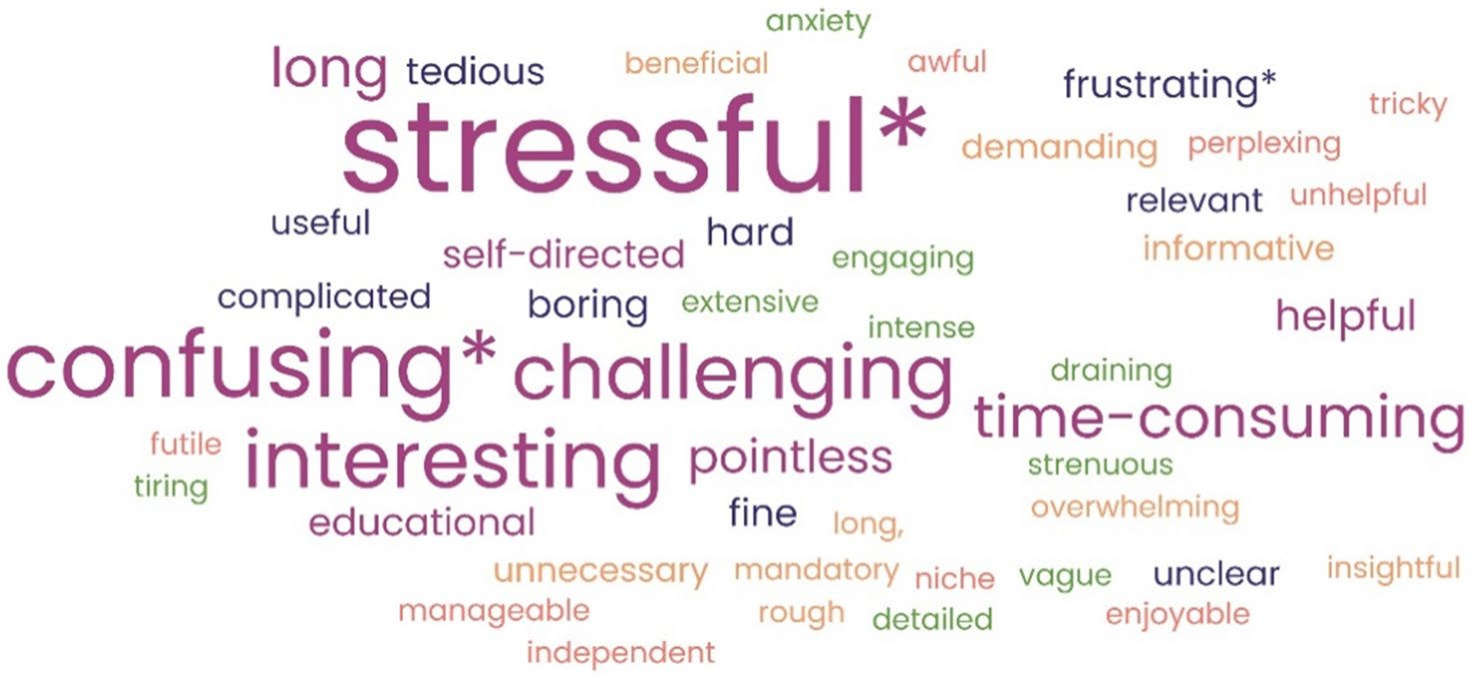
Word cloud showing the words used by students to describe the research project. *The use of that word or a derivative of that word (e.g., stress, stressful, stressed, etc.).

In the free text response items, the word ‘confusing’ or a derivative of this word (e.g., ‘confusion’) was used by 16 students (24%) (Figure 6) to describe their experience with the RP; this could relate to the perceived preparation for the project or to their guidance from supervisors regarding the project. Similar words such as ‘unclear’ and ‘vague’ were used to describe the project a further 3 times. Furthermore, 14 students (21%) left comments relating to a lack of preparation or resources for their project (Table 4).

#### 3.3.3 Theme C: Problems With Time Frame of the Project

Comments were left by a total of 16 fourth and fifth‐year students (24%) regarding the lack of time given to complete their project, with 8 comments being from 4th years and 8 being from 5th years. The majority of the comments acknowledged time as being the main barrier to the completion of their project, with quotes shown in Table 4.

#### 3.3.4 Theme D: Lack of Participation in Surveys

Students reported that a barrier to completion of their project was the lack of responses to surveys and questionnaires. Comments regarding the lack of responses were left by five students (8%).

#### 3.3.5 Theme E: Difficulties Obtaining Ethical Approval

Five students (8%) also commented on the difficulties with obtaining ethical approval as a barrier to completing their project in the timeframe. Students reported rejected ethical approvals delaying their project or the requirement for ethical approval putting them off completing primary research.

#### 3.3.6 Theme F: Improved Understanding of Research and Associated Skills

A total of 43 students (65%) said they had learnt more about research and/or the associated skills such as literature search strategies, applying for ethical approval, critical analysis and structuring research papers from completing their project. With 9 students (14%) stating in the free text items that they were in a better position should they want to conduct any research in the future. Whilst there were only 3 students (5%) who said that they did not feel as though they had learnt anything from their project. Responses from 6 students (9%) said that they felt that the research project would help their future practice and 7 (11%) said that the project had increased their interest in conducting further research or increased their interest in a specific topic or specialist area.

## 4 Discussion

This project aimed to explore undergraduate student views on a RP completed as part of their BDS degree, with the main objectives being to determine students' perceived preparedness for the project, to determine students' opinion of the importance of the project and to determine whether students or supervisors are driving the project. Undoubtedly, research and critical analysis form a vital part of the undergraduate dental curriculum, even for students who do not wish to pursue a career in academia, and the RP is a large part of the assessment method utilised at CU.

### 4.1 Timeline of Project

The difference in mean satisfaction score of the project between the 4th and 5th year students was not shown to be significant but overall there was a higher mean satisfaction score for 4th years. This difference in satisfaction could be related to the changes in the timeline of completion of the project for 4th years. For the 5th years, their projects were due in their 5th year of study and this is the same year as final exams, vivas, case reports and the national situational judgement test. In the fourth year, the main assessment is the RP with no end of year examinations. This gives the fourth years more time to focus on their project. Despite this, there were 16 comments regarding the issue of time for completing of the project in the free text response section, with students reporting that the ‘deadline too close to final exams’ as well as there being ‘no time in timetable dedicated’ to the RP and it was difficult ‘finding time alongside busy clinics’. Although the change in timeframe of the project has moved the deadline away from other examinations, there is still only 1 year in which to complete the project, which some students remarked put them off attempting primary research, and the 4th years still have lots of clinical time which they must balance with their projects. The undergraduate dental degree at CU is 5 years long which gives only a limited timeframe in which an RP could be completed and so extending the timeline of an RP may not be feasible alongside other examinations; there could, however, be specific time within the timetable during which an RP could be completed. Other institutions have projects which are either voluntary [10, 11] and/or completed over a number of years [11]. Other institutions have also found that the limited time in dental school is the main obstacle to students taking part in research [8], with this further complicated by the need to study and complete clinical work [1]. So whilst it may not be feasible to increase the timeline of the project, providing students with fixed time to complete their projects may improve students' perception of the project.

An overriding theme in the free text answers was the students' feeling of a lack of time to conduct an RP within the undergraduate curriculum. To improve the RP, it could be conducted over multiple years of study; however, this could then take up time required for other teaching, or a group format of the RP could be considered. Introducing a group format would allow students to support each other, and the labour of the project could be divided among students. Not only would this allow more work to be completed in the same time, but it would also encourage students to simultaneously develop skills such as teamwork and communication. If this was combined with each group receiving two or more supervisors, this would help with ensuring every student is adequately supported during their project. However, this may not be feasible as the marking of individual students becomes more challenging in a group format.

### 4.2 Inspiring Future Research

There were 15 students who reported in the free text items that their RP had increased or decreased their interest in a certain topic or interest in research itself. This is one of the purposes of education to expose students to different opportunities, whether clinical or non‐clinical; even if students decide they are not interested in research in the future, the exposure to research at an early stage of their career means that they have the opportunity to experience research and evaluation of data. A similar study of research projects at the Istanbul University Faculty of Dentistry showed that students who had completed a research project had a ‘better basis for postgraduate academic research’ [10].

### 4.3 Preparation for Research

The majority of students agree there were other opportunities within the curriculum to undertake research, but only 20% felt adequately prepared to begin an RP, and 56% thought insufficient information was given. Responses from free text items included statements such as ‘we did not get enough teaching/advice on this project’, ‘I feel as though I have not been properly taught how to write a research project and could have done with some more input prior’ and ‘had no idea how to write a research paper’. The data and statements suggest that further teaching regarding research and data evaluation in the context of their RP could have been beneficial. Although this teaching does form part of the UG degree, giving students more research and analysis teaching in the context of their project or during the completion of their projects may aid students' feelings of preparation and so increase satisfaction. There are other opportunities to undertake research during the undergraduate degree at CU; however, this is the only opportunity for students to decide their research topic and research type, that is, primary or secondary research. This lack of prior experience may contribute to the students' feelings of lack of preparation. The quantitative data reflected these findings, with students who felt more prepared enjoying the project more. There were also two comments stating that examples of previous RPs would have been beneficial. To ensure students feel prepared, there could be examples of different types of projects available for students, for example, both primary and secondary research examples. In addition to this, providing students with more teaching of research techniques throughout the duration of their project may help.

Students also reported not feeling adequately prepared to begin their project; research techniques are taught as part of the undergraduate curriculum at CU in the years prior to completion of the RP. However, the teaching of these topics in the context of the project could be beneficial to students to aid them in their project and serve as a reminder of previous teaching. Furthermore, providing students with examples of previous projects would help guide students in how to structure and lay out their project to conform to the criteria of the assessment. Students who feel more prepared are likely to perceive a greater benefit to the project.

### 4.4 Student–Supervisor Interactions

It is clear that there are contrasting experiences between students, with some reporting positive interactions with supervisors and others reporting negative interactions. The data show that those who had input into their project title enjoyed their project more. However, it would have been useful to add a further item about whether the students felt their supervisor had benefitted them with regard to the completion of their project. Unfortunately, it is challenging to ensure students have the same advice from and interactions with their supervisors. One student said they felt the RP ‘relies completely on your supervisor to guide you correctly’ which indicates the need for adequate training of all staff supervising projects. This reflects data from other studies which show that there is a negative implication on students' research experience due to a lack of supervisor mentorship [1, 11]. It also reflects the MLR, which showed that students who had more input into their title enjoyed the project more. Forty‐eight students agreed that they had had regular interactions with their supervisors, and 11 of these students said that it was their supervisor driving their interactions, whilst 23 said that they were the ones driving the interactions. This suggests that students are the driving factor in the completion of RPs. As the questionnaire did not ask students to identify their supervisor, it is impossible to determine what type of supervisor provides a better experience to students. However, differences in the roles of supervisors within the dental school may mean that they have different amounts of time to aid students. In addition to this, supervisors' opinions on the benefits of the RP may affect their interactions with students regarding the project. Furthermore, supervisors will all have different levels of experience within research, so those with more experience may be better able to support students with their projects. Supervisors should all be provided with adequate training so that supervisors with less research experience than their peers can provide the same level of support to their students. However, it is not possible to ensure that all supervisors have the same level of experience, but it can be ensured that all supervisors have adequate training. Follow‐up research to explore how prepared members of staff feel to support students through the conduction of an RP could identify areas of training that could be improved.

At CU, students who do not decide on a title by September of 4th year are assigned a supervisor who may be based in an area in which the student has no interest. It is important that students are guided in deciding a title to ensure that they are conducting research in an area that interests them and avoid assigning them a title they are not interested in; this will help to improve student engagement with projects.

### 4.5 Benefit of Project

As only 44% of students agreed that their RP would benefit them upon completion of their undergraduate degree, this suggests that students do not feel that the project is an important part of their education. MLR analysis showed that students who perceived a benefit to the project had a more positive experience with the project. It is therefore important for students to understand how undertaking research at an early stage in their career can benefit them in the future, for example, application to specialty training. It could be beneficial to include examples of how projects have been utilised beyond just passing the undergraduate degree within the briefing. However, as this survey was completed during the timeline of the project, the opinions of students may change once the project has been completed. Although it is important that research should help prepare students for graduation, the projects should also help students with their academic work whilst still an undergraduate. Research projects completed during undergraduate study help develop skills students will need upon graduation, such as interpersonal and critical analysis skills [11]. It has been shown in other studies that the academic performance of dental students who completed research projects during their degree is significantly better than those who have not [1, 10, 11].

Students' satisfaction scores with the RPs are affected by:

– Student input into the project title
– Perceived preparation for completing the project
– Understanding of the purpose of the project
– The belief that the project has helped develop their research skills.
– Student belief that the project will benefit them once they complete their undergraduate degree

Although conclusions can be drawn from the data provided in this project, the sample size is small (*n* = 66), which, coupled with the response rate of 52%, means that further research is required to reinforce these findings.

### 4.6 Limitations

This questionnaire did have drawbacks which are that the collection of data was anonymous, so it is possible that some students could have completed the survey twice, which could affect the results. In addition to this, the sample size was small (66), and ideally this survey would have a larger sample size, which could have been achieved by recruiting students who have graduated from CU to answer the questionnaire. Contacting students who have graduated from CU would enable further questioning as to whether they feel their projects have benefited their clinical practice or further research. In addition to the sample size being small, the response rate was only 52%, and further follow‐up emails may have been beneficial to increase the response rate. It has also been shown that generally people are more likely to share bad experiences than positive ones [15], and so the data from this questionnaire may be negatively skewed as not all students responded. The low number of responses may be due to students not feeling strongly either negatively or positively about the project, which is shown in the average rating of the project. Sending out more reminder emails to students may have encouraged more responses to the survey, which would have been beneficial.

Due to the restricted timeframe of this project, data collection was only carried out over 3 months, so there is insufficient evidence to determine if the perceived benefit of a RP increases as students' progress through the course. This is because the data was split into two groups: one of fifth years who had been completing their project for 484–513 days and one of fourth years who have been completing their project for between 141 and 170 days. Due to the data being limited to these two groups and there being no results from those who have already completed their project or not yet been introduced to the project, we cannot determine how the perceived benefit changes. Follow‐up research would be beneficial both upon completion of the project and once students have been practising dentists for 5 years to see if the students' opinion of the project changes and to see if students believe their project has helped them in their practice. Collecting information on the results of the project assessment could also be utilised to determine if the students who felt more prepared or had better interactions with their supervisor performed better overall. Further research would also show if students' opinions of the project changed throughout the duration of the project. Future research should also collect data on the roles of supervisors, for example, clinical or non‐clinical and previous research experience. This extra information would enable analysis of the benefit of different types of supervisors to student experiences and outcomes.

## 5 Conclusions

This project investigated the views of undergraduate students towards a RP which forms part of the undergraduate curriculum at CU. Overall, students had a positive opinion of the project, with the students who had more input into the project title and those who felt more prepared for the project enjoying it more. Despite the overall positive opinion of the RP, there is a large variation in students' experiences with the project. In order to ensure that all students receive the most benefit from a RP, there should be a more consistent experience for all students. One area in which student experiences differ is with their supervisors, who are the students' first point of contact for advice regarding their project. Extra training of all supervising staff with limited research experience should be implemented to ensure students all receive a similar experience. Consideration should be given to trialling a group project to improve experience.

Overall the RP implemented at CU has been shown to be viewed positively by students, but there is room for improvement of the project to ensure that all students benefit equally.

## Conflicts of Interest

The authors declare no conflicts of interest.

## Data Availability

The data that support the findings of this study are available on request from the corresponding author. The data are not publicly available due to privacy or ethical restrictions.

## Appendix A Questionnaire

1. What year of study are you currently in (year 4/year 5)
2. How do you agree with the following statements? (strongly disagree/somewhat disagree/neither agree nor disagree/somewhat agree/strongly agree)
  - I understand the purpose of the RP
  - I felt enough information was given prior to beginning the RP, e.g., guidance, resources, etc.
  - I have had other opportunities within the curriculum to undertake research
  - I think the research project will benefit me upon completion of my undergraduate degree
  - I understand why the project forms part of my education
  - I feel/felt adequately prepared to begin my RP
  - I feel the RP has helped me develop my research skills
  - I, rather than my supervisor, decided the focus of my RP
  - I have had regular interactions with my supervisor
  - My supervisor drove the method of interaction and organised our meetings
  - I had no input into the project title
  - I arranged meetings with my supervisor
3. Before beginning your RP, did you know what you wanted to focus your project on?
4. Please type 3 words which reflect your experience with the RP
5. Overall how would you rate your experience with the RP (1–10)
6. What have you learned from completing/working on this project
7. How do you think this has informed your future practice or learning?
8. What were/are the barriers and facilitators to you completing this project?
